# Mind-wandering Is Accompanied by Both Local Sleep and Enhanced Processes of Spatial Attention Allocation

**DOI:** 10.1093/texcom/tgab001

**Published:** 2021-01-15

**Authors:** Christian Wienke, Mandy V Bartsch, Lena Vogelgesang, Christoph Reichert, Hermann Hinrichs, Hans-Jochen Heinze, Stefan Dürschmid

**Affiliations:** 1 Department of Neurology, Otto-von-Guericke University, Leipziger Str. 44, 39120 Magdeburg, Germany; 2 Forschungscampus STIMULATE, Otto-von-Guericke University, Universitätsplatz 2, 39106 Magdeburg, Germany; 3 Department of Behavioral Neurology, Leibniz Institute for Neurobiology, Brenneckestr. 6, 39118 Magdeburg, Germany; 4 CBBS – center of behavioral brain sciences, Otto-von-Guericke University, Universitätsplatz 2, 39106 Magdeburg, Germany; 5 German Center for Neurodegenerative Diseases (DZNE), Leipziger Str. 44, 39120 Magdeburg, Germany

**Keywords:** high-frequency activity, local sleep, mind-wandering, N2pc, visual spatial attention

## Abstract

Mind-wandering (MW) is a subjective, cognitive phenomenon, in which thoughts move away from the task toward an internal train of thoughts, possibly during phases of neuronal sleep-like activity (local sleep, LS). MW decreases cortical processing of external stimuli and is assumed to decouple attention from the external world. Here, we directly tested how indicators of LS, cortical processing, and attentional selection change in a pop-out visual search task during phases of MW. Participants’ brain activity was recorded using magnetoencephalography, MW was assessed via self-report using randomly interspersed probes. As expected, the performance decreased under MW. Consistent with the occurrence of LS, MW was accompanied by a decrease in high-frequency activity (HFA, 80–150 Hz) and an increase in slow wave activity (SWA, 1–6 Hz). In contrast, visual attentional selection as indexed by the N2pc component was enhanced during MW with the N2pc amplitude being directly linked to participants’ performance. This observation clearly contradicts accounts of attentional decoupling that would predict a decrease in attention-related responses to external stimuli during MW. Together, our results suggest that MW occurs during phases of LS with processes of attentional target selection being upregulated, potentially to compensate for the mental distraction during MW.

## Introduction

Depending on the time spent awake and the richness of experiences rodents and humans enter local sleep-like states, which manifests both as high amplitude slow wave activity (SWA) in the delta/theta range (1–6 Hz) and brief neuronal silencing (Vyazovskiy et al. 2011). Local sleep (LS) refers to the occurrence of use-dependent, sleep-like slow oscillations in neuronal populations while being awake. These slow oscillations are temporally and spatially isolated and occur more often with sustained cortical use or prolonged wakefulness. On a neuronal level, LS is accompanied by neuronal silencing, i.e., short periods where neurons interrupt and then resume their firing pattern. The occurrence of these offline periods in behaviourally relevant cortical areas, e.g., motor cortex during a reaching task, can lead to performance errors (Vyazovskiy et al. 2011). Electrophysiologically, LS leads to localized peaks in slow wave oscillations (1–6 Hz, increased SWA), which served as a proxy for LS in previous human EEG studies (Murphy et al. 2011; Bellesi et al. 2014; Castelnovo et al. 2016). Recent intracranial recordings in nonhuman primates indicate that local epicortical high-frequency activity (HFA) consists of both infragranular single-unit and supragranular calcium-dependent dendritic processes (Leszczyński et al. 2020) and is a key marker of cortical activation (Ray et al. 2008). When local neuronal assemblies interrupt and then resume their firing patterns (LS), this interruption leads to a reduction of amplitude in the HFA range. In humans, increased SWA as well as worsened performance have been observed after extended practice and prolonged wakefulness (Hung et al. 2013; Bernardi et al. 2015). Phenomenologically, LS is assumed to unearth mind-wandering (MW) (Andrillon et al. 2019), during which attention shifts inwards to self-centered matters (Smallwood and Schooler 2006). MW encompasses that (i) we retrieve episodic memory while (ii) we are occupied with another task, and (iii) that we become aware of this episodic material (Smallwood and Schooler 2006). However, becoming aware of something cannot be confused with directing attention to it. But in practice it is challenging to disentangle consciousness and attention. Hence, in this study, we also pursue the question how tightly consciousness is coupled with attention or whether attention can be allocated elsewhere while we are conscious of a different matter. Andrillon et al. (2019) proposed that LS, occurring in attentional networks, might trigger the deactivation of those networks and the recruitment of the default mode network (DMN), which in combination then leads to MW. Whether LS indeed leads to MW is not clear. Here, we provide an initial study in which we test whether and how LS and MW are related. Both LS and MW increase behavioral errors (Carriere et al. 2008; Smallwood et al. 2008; Bernardi et al. 2015; Seli 2016; Leszczynski et al. 2017) promoting the prediction of perceptual and attentional decoupling (Schad et al. 2012; Christoff et al. 2016). Perceptual decoupling is attested by reduced electrophysiological responses to the perceptual input during MW (Smallwood et al. 2008; Kam et al. 2011, 2018; Christoff et al. 2016). However, reduced electrophysiological responses are often interpreted as evidence for a reduction in attention (“attentional decoupling” - Smallwood 2011; Schad et al. 2012) even if the respective EEG components are not associated with attention. Importantly, since off periods (LS and MW) during waking are potentially harmful (He et al. 2011; Kucyi et al. 2013; Yanko and Spalek 2014; Brandmeyer and Delorme 2018) the survival in general would be endangered if the brain´s need for rest is met entirely during waking at the expense of the ability to flexibly shift attention to key features in the environment (Vyazovskiy and Harris 2013). Here, we explicitly ask how the brain’s ability to shift attention varies during off periods (LS and MW) and whether MW leads indeed to an attentional decoupling.

To this end, we employ an established electrophysiological response attributed to the focusing of visual attention onto a target searched among distractors, the EEG component N2pc (Luck and Hillyard 1994a; Eimer 1996; Luck et al. 1997; Hopf et al. 2000; Mazza et al. 2009; Boehler et al. 2011). The N2pc is characterized by a more negative deflection at posterior EEG channels contralateral to the visual field in which the target was presented. Theoretically there are at least 2 principal scenarios which can be tested using the N2pc. On the one hand, the attentional decoupling account predicts that the N2pc as an index of attentional selection gradually decreases with MW. On the other hand, it could be hypothesized that the N2pc increases with MW. That is, MW and external distractors are assumed to share a common underlying mechanism (Forster and Lavie 2014; Unsworth and McMillan 2014) and the N2pc is known to increase with an increasing amount of distracting information (Mazza et al. 2009).

Using the high spatiotemporal and spectral resolution of magnetoencephalographic recordings (MEG) we investigated how cortical dynamics varied with self-reports ranging from being ON (uninterrupted focus on the external environment) to OFF (MW) the task. The task was to search for a color-defined pop-out (target) among task-irrelevant distractors. Moreover, we hypothesized that if associated with LS, MW leads to SWA and neuronal silencing. The latter we would expect to be reflected in a reduction in HFA (80–150 Hz). HFA is a correlate of population neural firing rate (Mukamel et al. 2005; Liu and Newsome 2006; Manning et al. 2009; Miller et al. 2009; Ray and Maunsell 2011) and preferred proxy for asynchronous areal activation (Miller et al. 2009; Privman et al. 2013; Coon and Schalk 2016; Kupers et al. 2017) and thus ideally suited to test neuronal silencing.

## Materials and Methods

### Participants

A total of 16 subjects (5 female, range: 18–39 years, *M*: 27.13, *SD*: 5.85) participated after providing their written informed consent. One subject who did not experience MW was excluded, resulting in 15 subjects in the final analyses. All participants reported normal or corrected-to-normal vision and none reported any history of neurological or psychiatric disease. All recordings took place at the Otto-von-Guericke University of Magdeburg and were approved by the local ethics committee (“Ethical Committee of the Otto-von-Guericke University Magdeburg”) and each participant was compensated with money. The sample size in our study was chosen according to previous studies examining the N2pc (e.g., Boehler et al. (2011): N = 15; Hopf et al. (2000): N = 12). Since our analytical approach for the N2pc is in part based on these studies, we required a similar sample size. Regarding the HFA, previous studies often used intracortical recordings. Here, sample sizes are typically limited to similar numbers of subjects (e.g., Tallon-Baudry et al. (2005): N = 14; Golan et al. (2016): N = 14).

### Paradigm

**Figure 1:**
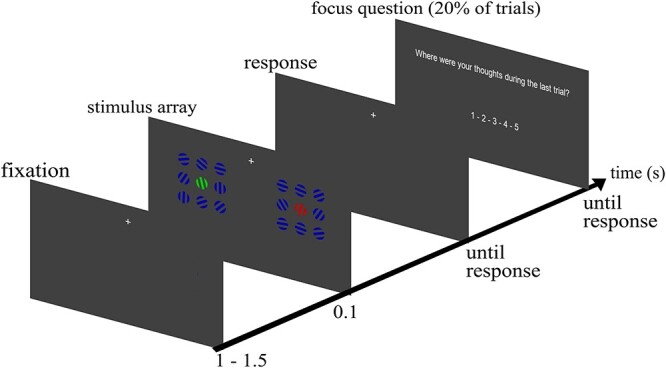
Single trial with focus question (see text for detail).

Participants were presented with a stimulus array of red, green, and blue grating patterns each consisting of 3 colored and 2 gray stripes viewed through a circular aperture (Fig. 1). The gray stripes matched the gray of the background. While either of the green and red gratings served as target, blue gratings always served as distractor items. Stimulus arrays consisted of 18 gratings arranged in 2 blocks of 9 gratings left and right below the fixation cross. Stimuli were placed below fixation since it has been shown that search displays evoke a stronger N2pc amplitude when displayed in the lower visual field (Luck et al. 1997; Hilimire et al. 2011). Participants were instructed to keep fixation on the fixation cross located at 1.9° visual angle (va) above the stimulus array. The size of each grating was 1.15° va, distance between single gratings (edge-to-edge) was 0.69° va. The left and right block of gratings each had a size of 4.83° by 4.83° va, the horizontal distance between both blocks (inner edges) amounted to 5.15° va. Diagonal distance between the fixation cross and the center of the nearest upper grating was 2.81° va. Target gratings could be tilted left or right in 10 steps of 1.5°, with the smallest tilt being 1.5° and the maximal tilt being 15° from the vertical axis. Orientation and tilt angle of the nontarget and distracter gratings varied randomly. Stimulus generation and experimental control was done using Matlab R2009a (Mathworks, Natick, USA) and the Psychophysics Toolbox (Brainard 1997; Pelli 1997; Kleiner et al. 2007). Colors were matched for isoluminance using heterochromatic flicker photometry (Lee et al. 1988).

### Procedure

At the beginning of each of the 12 blocks, participants were instructed to attend either only to the red or green grating and report via button press toward which side it was tilted (left: index finger, right: middle finger of the right hand). Target color assignment alternated blockwise. In blocks with the red grating as target the green grating served as nontarget which had to be ignored and vice versa. The target could appear at each of the 18 locations. The location of the nontarget was constrained to the mirrored location in the opposite grating block to keep equal distances to the fixation cross for both target and nontarget gratings. Each trial started with a fixation period of 1250 ms (±250 ms) before the stimulus array was presented for 100 ms. Participants were asked to respond as fast and accurately as possible. Afterwards the next trial started. The experiment started with a training block of 20 trials to familiarize participants with the procedure. After 20 consecutive trials, a blinking pause allowed participants to blink and rest their eyes. These pauses lasted 7 s. Each block consisted of 100 trials.

### Experience Sampling

Throughout the experiment we presented thought probes in pseudorandomly chosen trials (20% of all trials) asking participants to rate their attentional focus, in the single trial immediately preceding the probe, on a 5-point scale from 1 (“thoughts were anywhere else”—OFF) to 5 (“thoughts were totally at the task”—ON). The experience sampling approach allows the analysis of only a limited number of trials since more focus queries would prevent MW. Responses to focus questions were given with all 5 fingers of the left hand (thumb: 5, index finger: 4, middle finger: 3, ring finger: 2, little finger: 1). The probes were presented following orientation discrimination, with the restriction that 2 probes were separated by a minimum of one intervening search trial (this minimal distance of one intervening trial between probes occurred only for 7% of the focus queries). The probes were initiated by an auditory stimulus (500 Hz, ca. 85 dB for 200 ms). To increase statistical power, we grouped the 5 MW ratings in 3 groups of mental state (OFF: 1&2, MID: 3, ON: 4&5). Statistical analyses between mental states were performed on this subset of trials immediately preceding the focus query. Note that an increased number of thought probes (>20%) would lead to a decrease in time between single probes. This would leave little to no time for participants to let their minds wander, especially since the single trial duration in our experiment was only a few seconds.

### MEG Recording

Participants were equipped with metal-free clothing and seated in a dimmed, magnetically shielded recording booth. Stimuli were presented via rear projection onto a semi-transparent screen placed at a viewing distance of 100 cm in front of the participants with an LCD projector (DLA-G150CLE, JVC, Yokohama, Japan) that was positioned outside the booth. Responses were given with the left and right hand via an MEG compatible LUMItouch response system (Photon Control Inc., Burnaby, DC, Canada). Acquisition of MEG data was performed in a sitting position using a whole-head Elekta Neuromag TRIUX MEG system (Elekta Oy, Helsinki, Finland), containing 102 magnetometers and 204 planar gradiometers. Sampling rate was set to 2000 Hz. Vertical EOG was recorded using one surface electrode above and one below the right eye. For horizontal EOG, one electrode on the left and right outer canthus was used. Preparation and measurement took about 2 h.

### Preprocessing and Artifact Rejection

We used Matlab 2013b (Mathworks, Natick, USA) for all offline data processing. The 102 magnetometers were involved in our analyses. All filtering (see below) was done using zero phase-shift IIR filters (fourth order; filtfilt.m in Matlab). First, we filtered the data between 1 and 200 Hz. To discard trials of excessive, nonphysiological amplitude, we used a threshold of 3pT, which the absolute MEG values must not exceed (−1 to 2 s around stimulus onset—sufficiently long to prevent any edge effects during filtering). We then visually inspected all data, excluded epochs exhibiting excessive muscle activity, as well as time intervals containing artifactual signal distortions, such as signal steps or pulses. We refrained from applying artifact reduction procedures that affect the dimensionality and/or complexity of the data like independent component analysis. Time series of remaining trials were used to characterize HFA (80–150 Hz), SWA (1–6Hz), and the N2pc (1–30 Hz, main frequency range for cognitive event-related-potential (ERP) components, see Luck (2014)). Resulting time series were used to characterize brain dynamics over the time course of visual target detection. Each trial (−1 to 2 s around stimulus onset) was baseline corrected relative to the 100 ms interval prior to the stimulus onset.

### Statistical Analysis

Statistical analyses between mental states were performed on the trials immediately preceding the probe. Under the assumption that MW might comprise several trials, the focus query could have interrupted participants in the beginning, the middle, or at the end of an MW episode. Hence, including more than the trial directly preceding the focus question would have weakened the separation of mental states. To determine statistical significance, we compared each statistical parameter against a surrogate distribution, which was constructed by randomly yoking labels of the trials and repeating the analysis of variance (ANOVA), t-tests, and calculation of Pearson’s correlation coefficient. Consequently, reported *P* values represent the statistical significance relatively to the constructed surrogate distribution. We tested for statistically significant temporal intervals in 4 analyses: stimulus response of HFA, difference of the HFA between mental states, the N2pc, and difference of the N2pc between mental states. We considered only intervals with consecutive sample points exceeding 10 ms as significant (see Maris and Oostenveld (2007)). To correct statistical significance for multiple comparisons we applied Bonferroni correction. Since activity at each time point t linearly depends on activity at time point t-1, 2 adjacent tests cannot be regarded as independent. Hence, we determined how many individual components are contained in both the grand average HFA and N2pc and corrected the alpha value by the number of components that significantly explained variance. We carried out a principal component analysis (PCA) and determined the eigenvalues of the resulting components. Components with an eigenvalue larger than 1 were considered to explain a significant amount of variance within our data. In the HFA activity we found 5 and in the N2pc 4 individual components. Hence, the corrected *P* value for the HFA is 0.05/5 = 0.01 and for the N2pc 0.05/4 = 0.0125.

### I – Behavioral Results

We tested whether the ratio of ON and OFF ratings changed across the experiment to rule out the possibility that changes in cortical dynamic are a result of a change across the experiment and not of fluctuations of the mental state throughout the experiment. We divided the 12 experimental blocks in 4 parts by averaging ratings in 3 consecutive blocks since individual subjects did not make use of each of the 5 ratings in single blocks and compared the number of ON and OFF ratings across these 4 parts with a 4 × 2 ANOVA with the factors block (I, II, III, and IV) and mental state (ON vs. OFF).

Performance, measured as percent correct responses, was averaged across tilt angles for each subject and compared between mental states with a one-way ANOVA. Performance during focus trials was then correlated with N2pc amplitude (see below) to test whether N2pc strength predicts performance.

Reaction times (RTs) were grouped for the 3 mental states and averaged across subjects. The averaged RTs where then compared using a one-way ANOVA with the factor mental state (OFF, MID, ON).

### I‌I – HFA Response (Neuronal Silencing)

We then obtained the HFA response. For each trial we band-pass filtered each magnetometer´s time series in the broadband high-frequency range (80–150 Hz). We obtained the analytic amplitude ${A}_f(t)$ of this band by Hilbert-transforming the filtered time series. In the following, HFA refers to this Hilbert transform. We smoothed the HFA time series such that amplitude value at each time point *t* is the mean of 25 ms around each time point *t*. We then baseline-corrected by subtracting from each data point the mean activity of the 100 ms preceding the stimulus onset in each trial and each channel. Afterwards, we identified stimulus-responsive channels showing a significant (compared to an empirical distribution, see below) amplitude modulation in the HFA following the onset of the visual search array. Since we expected an HFA amplitude modulation within the first 300 ms following the stimulus presentation, we first calculated the average HFA modulation, averaged across the 300 ms following the stimulus onset, from which we then subtracted the baseline activity preceding the stimulus onset. Second, after stimulus-responsive channels were determined, a one-way ANOVA (OFF, MID, ON) was conducted at each time point between 100 ms prestimulus and 600 ms poststimulus to test for HFA differences between mental states. To facilitate interpretability, we report F-values after stimulus presentation. The F-value of the main effect “mental state” parameterizes neuronal silencing in the HFA response, with high F-values indicating a large difference in HFA amplitude between mental states. To set a threshold for significant difference, an empirical distribution of the main effect was constructed by randomly reassigning the labels (OFF – MID – ON) to the single trials in 1000 permutations. Peak responses (maximal average HFA response following stimulus onset) in each of the mental states were compared against a surrogate distribution. In each iteration, time series of each channel were circularly shifted time series of participants between -500 and 300 ms separately, and new (surrogate) trial averages were calculated. From these trial averages we calculated the peak value in the time range of 0 to 300 ms following stimulus onset. Mental states exceeding the 97.5th percentile were classified as showing significant HFA modulation.

The HFA is a frequency band, whose amplitude is modulated by stimulus presentation both in the auditory (Crone et al. 2001) and visual modality (Lachaux et al. 2005). Hence, the HFA is a stimulus-responsive band. The usual 2-step approach is to (i) assess stimulus-responsive channels and then (ii) test for condition differences. The prediction from recent MW literature is that the sensory representation of an onsetting stimulus is low when subjects report that their minds wandered. Using the HFA, we can test which regions are stimulus-responsive. Slow wave oscillations, in contrast, are instantaneous in the sense that they occur locally but might travel across cortical regions during sleep. These occasionally appearing SWA peaks are different from ongoing activity that should be modulated by stimulus onsets. Hence, SWA-peaks are not assumed to carry stimulus information. Instead, synchronized occurrences of OFF periods result in the high-amplitude electro- or magnetoencephalogram (EEG/MEG) slow waves that are typical for early, nonrapid eye movement sleep. The electrographic manifestation of sleep—high-amplitude EEG/MEG slow waves—arises from such synchronized alternation between on and off periods across large cortical neuronal populations.

### I‌II – High-Amplitude Slow Wave Oscillation

For each trial we band-pass filtered each magnetometer´s time series in the frequency range of slow wave oscillations (1–6 Hz) and z-scored the obtained analytic amplitude ${A}_f(t)$ of this band by Hilbert-transforming the filtered time series. In the following text, SWA refers to this Hilbert transform. We used z-scoring for the SWA for 2 reasons. First, SWA peaks are single temporally and spatially isolated events (Vyazovskiy et al. 2011), while the HFA is an ongoing time series (Crone et al. 2001). Second, unlike the HFA, the SWA is not stimulus-responsive. SWA pattern can occur even in the baseline period. In contrast to the HFA, we did not expect the number of SWA peaks to be modulated by the stimulus onset. Hence, the z-score method allows to assess the local occurrence of SWA independently of stimulus onset across the entire recording time. We then counted the number of peaks of the SWA defined as local maxima exceeding 3 SD in each trial at each channel in the time from 500 ms prestimulus to 500 ms poststimulus. Next, we identified channels with a high number of SWA peaks. To account for the occurrence of SWA peaks local in time, a surrogate distribution was constructed by randomly exchanging channel labels in each subject and calculating new (surrogate) channel averages across participants. In each of 1000 iterations we randomly exchanged channel labels in each subject and new (surrogate) channel averages were calculated across participants. Channels exceeding the 97.5th percentile of the channel-specific surrogate distribution were classified as showing a significant SWA modulation (SWA channels). The number of SWA peaks was averaged separately for the 3 mental states across SWA channels in each participant. We then carried out a one-way ANOVA with factor mental state (OFF – MID – ON) at each time point, with single participants as random variable. The F-value of the main effect “mental state” parameterizes the occurrence of SWA with high F-values indicating a large difference in the number of SWAs between mental states. To set a threshold for significant difference, an empirical distribution of the main effect was constructed by randomly reassigning the labels (OFF – MID – ON) to the single trials in 1000 permutations.

The rationale for the different analytic approaches for SWA and HFA, even though they reflect presumably similar processes, is the following: Modulation of the HFA is usually assessed as its variation across time. In contrast, SWA are single events local in time. The difference in analytic approaches is also due to the fact that low-frequency characteristics can be detected easier in macroscopic recordings than high-frequency patterns which is why they can be localized more feasible in time (i.e., as single events in time). However, both measures are strongly related since an increase of SWA in the rodent’s LFP is paralleled by neuronal silencing. In our study we can assess SWA but not multiunit activity that could directly index neuronal silencing. The neural signature closest to the MUA, however, is the HFA, which has been regarded a good measure of neuronal spiking (Liu and Newsome 2006; Berens et al. 2008) and consists of both infragranular single-unit and supragranular calcium-dependent dendritic processes (Leszczyński et al. 2020). This is also consistent with the idea that HFA reflects aggregated local neuronal output (Buzsáki et al. 2012) due to reliably high correlations between HFA and multiunit activity. Hence, the HFA became a classical indicator of cortical activation.

### IV – N2pc

To assess the allocation of spatial attention, we employ the so-called N2pc, which is a marker of attentional selection in visual search paradigms (Luck and Hillyard 1994a; Eimer 1996; Luck et al. 1997; Hopf et al. 2000). The N2pc is an event-related component of the EEG and MEG response that is elicited contralateral to the target when subjects covertly (i.e., without eye-movements) shift their spatial attention to the respective target presented in the left or right visual field. Specifically, shifting the focus of attention to the left visual hemifield will lead to an enhanced response—typically around 200–300 ms after stimulus-onset—at right-hemisphere sensors and vice versa. Importantly, a stronger N2pc (higher amplitude) is associated with a stronger focusing to the respective target item and/or better suppression of surrounding distractor items (Luck et al. 1997; Mazza et al. 2009). The N2pc is recorded at sensors showing a maximum difference in response to left versus right visual field targets, typically at parietal/occipital recording sides. For EEG, there is usually a single maximum (negativity) contralateral to the target. For MEG, the respective dipole creates both an efflux and an influx maximum contralateral to the target that will be combined (Hopf et al. 2000; Boehler et al. 2011). The N2pc can then be displayed as the respective left-minus-right difference waveform with the signal often being averaged across both hemispheres for simplification (i.e., only one single waveform for the N2pc combining attended left and right visual field targets) (e.g., Mazza et al. 2009; Lagroix et al. 2015; Donohue et al. 2018). Extraction of the N2pc waveform was adapted from Boehler et al. (2011). For each participant, 4 channels were selected. One in each hemisphere reflecting the efflux maximum and one in each hemisphere reflecting the influx maximum. Selection of channels was limited to an occipital-parietal region of interest (ROI) which is in line with the N2pc ROI in the previous literature (e.g., Hopf et al. 2000; Boehler et al. 2011; Donohue et al. 2016). Efflux and influx channels of both hemispheres were combined by subtracting the signal of the influx channel from the signal of the efflux channel. To extract the N2pc, we subtracted this combined signal for targets in the right visual field from the combined signal for targets in the left visual field, again separately for both hemispheres. The final N2pc waveform was generated by averaging together the N2pc generated over the left and right hemisphere. In the next step we tested whether the N2pc was significantly elevated over baseline. We baseline-corrected the N2pc time series of each subject by subtracting from each data point the mean activity of the 200 ms preceding the stimulus onset. We then tested whether the average N2pc shows a significant (compared to an empirical distribution, see below) amplitude modulation following the onset of the visual search array. We first calculated the average activity modulation ${\overline{A}}_{\mathrm{N}2\mathrm{pc}}$ averaged across the 200–300 ms following the stimulus onset from which we subtracted the baseline activity ${\overline{B}}_{\mathrm{N}2\mathrm{pc}}$ preceding the stimulus onset. The difference between $\overline{B\ }$ and $\overline{A}$ was compared against a surrogate distribution. In each iteration, time series of each subject were circularly shifted between −500 and 300 ms separately, and new (surrogate) trial averages ($\overline{B\ }\mathrm{and}\ \overline{A}\Big)$were calculated. Time points exceeding the 97.5th percentile of the channel-specific surrogate ${\overline{A}}_{N2 pc}-{\overline{B}}_{N2 pc}$ distribution were classified as showing a significant N2pc modulation following stimulus onset. The first time point of significant N2pc modulation in each subject was used as N2pc onset. Using a time point-by-time point ANOVA between -100 and 600 ms with the factor mental state (OFF, MID, ON) we tested whether the N2pc differs between focus conditions. The F-value of the main effect “mental state” parameterizes the variation of the N2pc as a function of mental states with high F-values indicating a large difference in N2pc amplitude between mental states. To set a threshold for significant difference, an empirical distribution of the main effect was constructed by randomly reassigning the labels (OFF – MID – ON) to the single trials in 1000 permutations.

**Table 1:** Mean number and percentage of thought probes, categorized as ON, MID, or OFF

	Mean %	SD %	Mean N	SD N
ON	51.25	27	107.14	46.75
MID	33.1	18.7	76	42.64
OFF	15.67	16.8	34.92	36.27

### V – Local Sleep-N2pc Correlation

First, HFA and N2pc onset times were compared via t-test to analyze temporal discrimination between both. Second, to examine the interaction between HFA and N2pc over the different mental states, HFA and N2pc time series were averaged separately for the 3 mental states in each participant for the interval between onset and offset (interval between significant elevation over baseline). We then carried out a 2-way ANOVA with factor MEG response (N2pc – HFA) and mental state (OFF – MID – ON) at each time point, with single participants as random variable. Third, for each mental state N2pc (averaged across the interval of significant amplitude modulation for all trials) was correlated with HFA response (averaged across the interval of significant amplitude modulation for all trials). The resulting Pearson’s correlation values were tested against a surrogate distribution. This surrogate distribution was constructed by randomly assigning the HFA values of each participant with the N2pc values from another participant in 1000 iterations.

## Results

### I – Behavioral results

**Figure 2:**
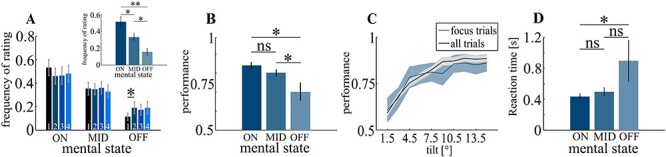
Behavioral data, A: participants made more ON and MID than OFF ratings (small inset). Only between the first and the second quarter of the experiment was a significant increase in OFF ratings, which then remained constant. Numbers at the bottom of bar graphs indicate the corresponding quarter (i.e., first, second, third or fourth) of the experiment. B: subjects made more errors during OFF trials than during ON and MID trials. C: Performance was lowest at small tilt angles and increased with increasing angles. This pattern was identical for all trials (black) as well as for the trials in which a thought probe was presented (blue), irrespective of mental state. D: Reaction times were significantly longer in OFF vs. ON trials. The error bars and shaded areas represent the standard error of the mean (SEM). ^*^  *P* < 0.05, ^*^^*^P < 0.01.

Excluding times for individual pauses, thought probes were presented on average every 10.34 s (SD = 5.09; range: 3.7–29.6 s). MW ratings differed in frequency (*F*_2,42_ = 10.11, *P* < 0.001; ON 51.25% (*SD:* 27%), MID 33.1% (*SD:* 18.7%), and OFF 15.67% (*SD:* 16.8%); Table 1) with more ON than MID ratings (*t_14_* = 2.21, *P* = 0.035) and more MID than OFF ratings (*t_14_* = 2.56, *P* = 0.016). The ratio of ratings did not vary across blocks: main effect of block (*F*_3,112_ = 0.03, *P* = 0.99) and interaction (*F*_3,112_ = 0.6; *P* = 0.6) were not significant (Fig. 2*A*). While ON ratings did not vary across blocks (all *P*’s > 0.1), OFF ratings increased from block I to II (*t_14_* = 2.5; *P* = 0.02) but remained constant afterwards. Performance varied with mental state (*F*_2,42_ = 5.14, *P* = 0.01) with worse performance during OFF trials (*M*: 70.2%, *SD*: 18.8%) than during MID trials (*M*: 80.2%, *SD*: 7%; *t*_14_ = 2.62, *P* = 0.01) or ON trials (*M*: 84.7%, *SD*: 7%; *t*_14_ = 2.09, *P* = 0.03). No differences were observed between ON and MID trials (*t*_14_ = 1.76, *P* = 0.1; Fig. 2*B*). Performance varied also as a function of tilt angle. Participants made more errors at small angles and performance increased fast with increasing angles. To increase statistical power, we averaged trials across all tilt angles (Fig. 2*C*). Also, reaction times differed significantly between mental states (*F*_2,42_ = 2.75 *P* = 0.003) with longer RTs during OFF (*M*: 898 ms, *SD*: 1028 ms) compared with ON (*M*: 433 ms, *SD*: 146 ms; *t_14_* = 1.72, *P* = 0.04), a trend of statistical significance between OFF and MID trials (*M*: 489 ms, *SD* 212 ms; *t*_28_ = 1.48, *P* = 0.07), but no differences between ON and MID trials (*t*_28_ = 0.87, *P* = 0.38; Fig. 2*D*).

### I‌I – HFA Response (Neuronal Silencing)

**Figure 3:**
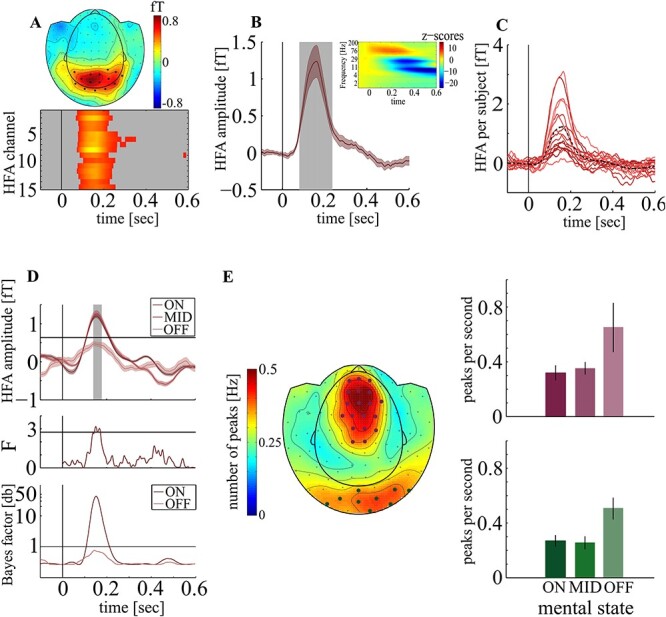
HFA A: Grand Average event related magnetic field (ERMF, 80–150 Hz) averaged across all focus trials and subjects between 0 and 300 ms poststimulus (top) shows 15 occipital sensors with significant response after stimulus onset. HFA onset and time course (bottom) are highly similar. B: Averaged across all trials and subjects, we found an HFA between 81 and 234 ms poststimulus (gray shaded area). The inset shows the time frequency representation averaged across all 15 MEG sensors. C: HFA response averaged across significant sensors for each subject. The dotted black line represents average across subjects. D top: HFA for each mental state, averaged across subjects. Gray inset represents time of significant differences in amplitude between mental states. The horizontal line represents critical peak amplitude modulation. D middle: Time course of F-values. The horizontal line represents critical F-value for statistical significance. D bottom: Bayes factor for amplitude modulation above baseline for ON and OFF condition. E: 28 Sensors showed significant SWA (left). The number of SWA peaks in occipital sensors (green, lower right) was significantly elevated during OFF trials (red: frontal sensors). The vertical lines represent stimulus onset. The shaded areas around curves represent SEM.

A total of 15 occipital magnetometers showed stimulus response in the HFA between 81 and 234 ms poststimulus (HFA_max_ = 1.24fT at 161 ms, *P* < 0.001, Fig. 3A,B,C). The HFA differed between mental states between 145 and 171 ms poststimulus (F_crit_ = 2.74; *F*_max_ = 3.18 at 151 ms, *P* = 0.02, Fig. 3*D*) with smaller HFA in OFF (*M*: .47fT, *SD*: .93fT) versus ON (M: 1.24fT, SD: .82fT; *t_14_* = 2.16, *P* = 0.02) and versus MID trials (*M*: 1.25fT, *SD*: 1.28fT; *t_14_* = 2.04, *P* = 0.03) but no difference between ON and MID (*t_14_* = 0.53, *P* = 0.69). Importantly, in contrast to ON (critical peak amplitude = 0.63fT, HFA_max_ = 1.29fT at 149 ms; *P* < 0.001) and MID trials (HFA_max_ = 1.33fT at 152 ms; *P* < 0.001), HFA did not show a significant peak response in OFF trials (HFA_max_ = 0.5fT at 151 ms, *P* = 0.15). We then tested whether the HFA is simply not different from but equals baseline activity. Specifically, we estimated the Bayes factor (BF) to determine the amount of evidence for a change over baseline (amplitude values across all time points and subjects in the 100 ms prior to stimulus onset). The BF was estimated at each time point separately for the ON and OFF condition. We found no evidence for the H_1_ and therefore no evidence for a change (BF_max_ = 0.76 at 142 ms) in the OFF condition. But strong evidence in the ON condition (BF_max_ = 43.01 at 152 ms; see Fig. 3*D*).

### I‌II – High-Amplitude Slow Wave Oscillations

A total of 28 MEG sensors covering a frontal-parietal (N_crit_ = 0.3 Hz; N_SWA_ = 0.43 Hz; *P* < 0.0001) and an occipital channel cluster (N_SWA_ = 0.38; *P* < 0.0012, Fig. 3*E*) showed a significant number of SWA. In frontal-parietal sensors we observed a trend toward differences in frequency of SWA between mental states (*F*_2,42_ = 2.7; *P* = 0.07, Fig. 3*E*), but a highly significant difference in occipital sensors (*F*_2,42_ = 5.9; *P* < 0.0001, Fig. 3*E*) with more SWA peaks in OFF (N_SWA_ = 0.51) versus ON (N_SWA_ = 0.27; *t_14_* = 3.4; *P* = 0.004) and versus MID trials (N_SWA_ = 0.25; *t_14_* = 2.6; *P* = 0.02) in theboccipital region.

### IV – N2pc

**Figure 4:**
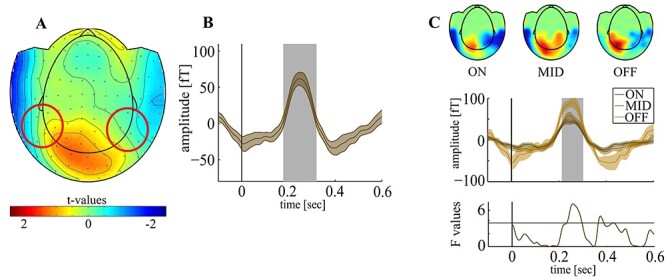
N2pc A: Grand average event related magnetic field (ERMF; 1–30 Hz) averaged across analyzed trials between 200 and 300 ms poststimulus. The circles represent probable location of underlying dipoles. B: N2pc averaged across analyzed trials and subjects. We found a significant N2pc between 179 and 319 ms poststimulus (gray inset). C top: Grand average ERMF between 200 and 300ms for the 3 mental states. Please note that the topographical field distribution and sensor locations are well in line with the literature with an occipito-temporal maximum evolving between 200 and 300 ms (Hopf et al. 2000; Boehler et al. 2011) and are highly consistent across the mental states. C middle: N2pc for each mental state, averaged across subjects. We found significant differences in N2pc amplitude between mental states (gray inset) between 213 and 298 ms poststimulus. C bottom: time course of F-values. The horizontal line represents critical F-value. The vertical lines represent stimulus onset. The shaded areas around curves represent SEM.

Attentional target selection elicited an N2pc between 179 and 319 ms poststimulus (N2pc_crit_ = 4fT, N2pc_max_ = 61.7fT at 258 ms, *P* < 0.001; Fig. 4A,B) with no differences between hemispheres (*t*_crit_ = ±2.74, *t*_max_ = -1.74 at 71 ms, *P* = 0.94). The N2pc differed between mental states between poststimulus (*F*_crit_ = 3.53, *F*_max_ = 7.62 at 256 ms poststimulus, *P* < 0.001; Fig. 4*C*) with a larger amplitude in OFF (*M:* 78.69fT, *SD:* 46.16) versus MID (*M:* 50.65fT, *SD:* 28.89; *t_14_* = 3.44, *P* = 0.01) and versus ON (*M:* 38.82fT, *SD:* 19.73; *t_14_* = 4.1, *P* = 0.002) but no significant difference between ON and MID trials (*t_14_* = 0.39, *P* = 0.69).

### V – Local Sleep-N2pc Correlation

**Figure 5:**
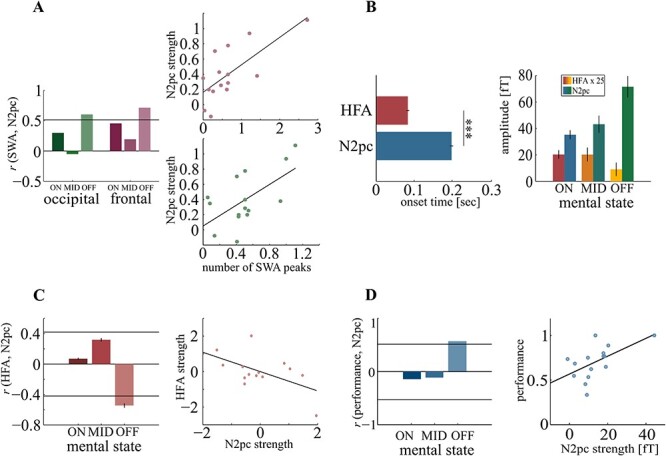
Local sleep-N2pc correlation A: Correlation between SWA count and N2pc amplitude was significant only during OFF trials. The horizontal line represents critical correlation value (left). Scatterplots showing the correlation between SWA count and N2pc for OFF trials in frontal (red, upper) and occipital sensors (green, lower right). B: Onset times for HFA and N2pc differed significantly (left). Average HFA and N2pc amplitude for each mental state. Note that the HFA is scaled up in this plot to compensate for lower amplitudes (right). C: Correlation between HFA and N2pc reached significance only during OFF trials. The horizontal lines represent critical correlation values (left). Scatterplot showing the correlation between HFA and N2pc during OFF trials (right). D: Correlation between performance and N2pc reached statistical significance only during OFF trials. The horizontal lines represent critical correlation values (left). Scatterplot showing the correlation between performance and N2pc strength during OFF trials (right). The error bars represent the SEM. ^*^^*^^*^P < 0.001.

The number of SWA peaks correlated with the N2pc in OFF trials both in the fronto-parietal and the occipital channel cluster (rcrit = 0.53, fronto-parietal: r = 0.71; *P* = 0.0044; occipital: r = 0.6; *P* = 0.014) but not in ON (fronto-parietal: *r* = −0.29, *P* = 0.13; occipital: r = 0.45, *P* = 0.04) or MID trials (fronto-parietal: *r* = −0.04, *P* = 0.56; occipital: r = 0.19, *P* = 0.22; Fig. 5*A*). Note that peaks of SWA and the N2pc were both well separable from each other, though their topographies did show some overlap at occipital sensors. Specifically, the SWA peaks were evenly distributed across time intervals before and after stimulus onset and were not time-locked to the N2pc, which could have confused measures of SWA peaks with the occurrence of the N2pc amplitude. A respective analysis was performed for the occipital SWA peaks. We compared the number of SWA occurrences in the N2pc interval (200–300 ms) against that of a prestimulus interval (-100–0 ms) and that of a later poststimulus interval after the N2pc (400–500 ms) and found no difference in neither comparison (t_14_ = 1.66; *P* = 0.12 and t_14_ = 0.93; *P* = 0.37). That is, the SWA peaks do not correlate in time with stimulus onset or N2pc emergence.

Importantly, the HFA (reflecting initial visual response) showed a significantly earlier onset than the N2pc (HFA: 83 ms poststimulus, SD: 14 ms; N2pc: 198 ms poststimulus, SD: 17 ms; *t_14_* = 20.1, *P* < 0.001, Fig. 5*B*, left). Average HFA and N2pc showed a strong interaction with mental states with the N2pc increasing with decreasing HFA (*F*_2,87_ = 11.17, *P* < 0.001; Fig. 5*B*, right). Similarly to SWA, only in OFF trials HFA correlated with the N2pc (*r*_crit_ = ±0.42, *r* = −0.54, *P* = 0.04), indicating that a low HFA amplitude is associated with an increased N2pc amplitude but not in ON (*r* = 0.07, *P* = 0.71) or MID trials (*r* = 0.31, *P* = 0.27, Fig. 5*C*). This enhancement of the N2pc appeared to be behaviorally relevant as in OFF trials, the N2pc was correlated to performance (*r*_crit_ = ±0.53, *r* = 0.57, *P* = 0.02) but not in ON (*r* = −0.14, *P* = 0.29) or MID trials (*r* = −11, *P* = 0.33; Fig. 5*D*).

## Discussion

We examined how indicators of LS, cortical processing, and attentional selection change during MW. Participants performed a visual search paradigm, yielding robust increases in the HFA response in occipital MEG sensors, followed by the N2pc responses reflecting attentional target selection. The onset of the HFA increase in occipital MEG sensors was as early as ~90 ms and depended on how focused participants were on the task. Specifically, under MW, the HFA response was strongly decreased (i.e., no significant difference from baseline). But what caused the reduced HFA response during MW? If changes in the activation of the attention network would be the cause for the HFA reduction, we would also expect to observe a modulation at fronto-parietal sensors. Hence, we think that this very early occipital HFA reduction most likely corresponds with neuronal silencing (Vyazovskiy et al. 2011) reflecting local sleep. In parallel, the number of SWA periods increased with MW, consistent with participants experiencing phases of local sleep. In line with previous studies, the performance decreased under MW with manual reaction times being substantially prolonged. In contrast, neural markers of attentional selection were even more pronounced during MW and closely linked to behavioral responses. That is, even though low in performance during OFF trials, subjects that showed a higher N2pc amplitude performed better than those with a less pronounced N2pc. In general, processes of attentional target selection, as indexed by the N2pc, were rather increased during MW, potentially compensating for mental distraction.

Grating stimuli reliably evoked high-frequency activity in our noninvasive MEG recordings strongly resembling HFA responses in intracranial recording in early visual cortex with a modulation over baseline between 50 and 350 Hz, a fast increasing flank peaking around 200 ms, and a slowly decreasing flank in early visual cortex (Burke et al. 2014; Szczepanski et al. 2014; Golan et al. 2016, 2017; Gerber et al. 2017; Helfrich et al. 2018; Bartoli et al. 2019). The high similarity of the HFA response across subjects indicates that MEG in contrast to EEG can reliably pick up HFA responses to visual stimuli, which even has been shown at the single trial level (Westner et al. 2018).

The HFA reduction during MW might not result from attentional decoupling but rather reflects neuronal silencing. Previous studies showed reduced electrophysiological responses during MW (Christoff et al. 2016) potentially due to attentional decoupling during MW but without deciphering the causal relation between MW and reduced cortical responses. The authors assumed that MW attenuates the cortical response (Christoff et al. 2016)—the HFA—since attentional resources are shifted inwards (Smallwood and Schooler 2006) in line with an attentional decoupling account. However, we hypothesize that participants experience MW, since use-dependent neuronal silencing reduces sensory representation of the visual environment in the first place. First, any attentional reduction of the HFA should also predominantly be found in fronto-parietal structures (Szczepanski and Kastner 2013; Szczepanski and Knight 2014; Perrone-Bertolotti et al. 2020) where we did not find any strong stimulus-driven modulation in our study. Second, and most importantly, attentional modulations of cortical responses are amply attested with a reduction of responses (Smallwood et al. 2008; Kam et al. 2011, 2018) often using a contrast between task relevant versus irrelevant stimuli (Müsch et al. 2014). But task-irrelevant stimuli still evoked a comparable HFA response even though smaller in amplitude. Also, in audition even though ignoring the stimulation and attending a second task, clear stimulus-driven responses can be seen in frontal and temporal cortex (Dürschmid et al. 2016). Hence, although modulated by attention, ERPs and HFA response in previous studies were preserved. In contrast, the here observed HFA increase in occipital MEG sensors was virtually absent under MW (no significant difference from baseline). Hence, we assume the strong reduction in HFA during MW is most likely not driven by attention but rather corresponds with neuronal silencing (Vyazovskiy et al. 2011) reflecting local sleep.

Importantly, only local sleep would potentially allow for independent regulation of attentional resources. A global state change, in contrast, would downregulate attentional resources concomitantly. Hence, the strong interaction between N2pc and HFA speaks in favor of the occurrence of brief periods of local sleep, which is typically observed for single units during NREM sleep (Vyazovskiy et al. 2011; Siclari et al. 2017) even in the absence of signs of drowsiness. The HFA, a localized index of functionally selective activity (Crone et al. 1998; Miller et al. 2007) and most likely reflecting multiunit activity, seems almost completely absent during MW in regions strongly responding to stimulation. In addition, in sleep-restricted humans, the waking EEG typically shows increased low-frequency power (SWA) reflecting the duration of prior wakefulness (Finelli et al. 2000; Leemburg et al. 2010; Vyazovskiy et al. 2011). Moreover, a homolog phenomenon to neuronal silencing can be observed in brain regions that were disproportionately used during waking (Rector et al. 2009) and involved in prior learning (Hung et al. 2013). Both strong signatures of local sleep—i.e., HFA reduction and SWA increase—did not overlap spatially but occurred locally (Bellesi et al. 2014), which points at different functions.

SWA could serve as a carrier wave that allows or drives the transfer of information between structures such as the hippocampus and neocortex. It occurred over centro-parietal, sensory, and motor area regions relative to the rest of the brain in a previous study (Castelnovo et al. 2016). In line with those results, we found an increase in centro-parietal and in occipital cortex. The parallel SWA increase between these regions argues strongly for a common plasticity-dependent component to sleep regulation (Murphy et al. 2011). Importantly, these signatures of local sleep occur even in subjects which are not sleep deprived (Quercia et al. 2018). The here observed SWA does probably indicate sleep need (Huber et al. 2004) but it varies locally in time, since subjective ON and OFF task ratings were evenly distributed across the entire experiment. Hence, we can rule out the possibility that the observed signatures of LS only increased with time and thus without any strong relation to MW.

Local sleep periods are of behavioral relevance since they are associated with cognitive lapses (Nir et al. 2017) that are marked by prolonged reaction times (Bernardi et al. 2015; Nir et al. 2017). The response time prolongation during such lapses probably arises due to reduced stimulus-triggered activity in visual areas causing a lower quality perceptual representation of the target stimulus (Weissman et al. 2006). Consistent with subjects experiencing attentional lapses, we also found reaction times to be substantially longer during MW. The observed motor slowing might in part explain behavioral errors in previous studies on MW as well. MW manifests behaviorally especially in highly automated task like reading or the Sustained-Attention-to-Response-Task (SART) (Smallwood et al. 2008; Seli 2016). Hence, behavioral decrements in SART experiments could result from a slowing of a general control of manual responses which could hypothetically be beneficial to prevent overhasty decisions when sensory evidence is low. Lower sensory evidence on MW trials would also be in line with an upregulation of the N2pc attention allocation response. That is, though low in performance, subjects with stronger N2pc perform better, underscoring the behavioral relevance of an upregulation of attentional resources to keep performing the task under MW.

Indeed, our major finding is that during local sleep the strength of SWA and neuronal silencing predicts how attentional reallocation is modulated. Previously, MW was found to positively correlate with task-irrelevant distraction indicating that MW reveals individual susceptibility to task-irrelevant distraction including both internal and external sources (Forster and Lavie 2014). Specifically, it was suggested that MW and external distraction reflect distinct, yet correlated constructs related to working memory (Unsworth and McMillan 2014). Hence, the N2pc increase is in line with previous studies showing that target-distractor disambiguation increases with distractor load (Mazza et al. 2009) and suggesting a stronger influence of distractors under momentary attention lapses (Weissman et al. 2006). These results indicate that MW does not inflict attentional decoupling (Smallwood and Schooler 2006). Given the earlier onset of HFA compared to the N2pc, the reduction in HFA during MW (worse stimulus representation) might consequently lead to the upregulation of the N2pc (more target enhancement and/or distractor suppression needed). Since experience sampling can only be applied in a subset of trials, a trial-wise measure of MW cannot be provided. Hence, we cannot dissolve the number of trials by which neuronal silencing is ahead the N2pc upregulation.

The N2pc was originally interpreted as suppression of distractors (Luck and Hillyard 1994b), but others argued that the N2pc reflects target selection (Eimer 1996) and is now considered a composition of overlapping processes of both target processing (target negativity, Nt) and distractor suppression (distractor positivity, Pd) (Hickey et al. 2009; Hilimire et al. 2012; Gaspar and McDonald 2014). Since we presented the target simultaneously with a color pop-out nontarget in the opposite visual field, both the target selection (Nt contralateral to the target) as well as distractor suppression (Pd contralateral to the pop-out nontarget) will contribute to the amplitude of the observed N2pc waveform. Importantly, we observed an enhanced N2pc when the subjects were in a state of MW. Since our stimuli always contained both laterally presented targets and distractors, we cannot unambiguously decide as to whether the enhanced N2pc was caused by a stronger target enhancement, increased distractor suppression, or both, or whether the N2pc would be rather generally suppressed in the focused state. In general, the N2pc component seems to strongly depend on stimulation parameters, showing larger activation differences between hemispheres when more than one item per visual field is presented, the discrimination task is more complex (e.g., a feature conjunction task), and the target is in the lower visual field (Luck et al. 1997). Hence, we chose our visual search display accordingly to maximize the observed N2pc amplitudes with the target being located in the lower visual field, multiple surrounding irrelevant distractor items, and a task requiring high spatial scrutiny. Most importantly, the target was always an easily detectable color pop-out item, requiring no time-consuming search process that might have smeared out N2pc responses over time. In fact, the N2pc was elicited at the expected time range of 200 ms irrespective of the mental state. That is, the initial target selection was not delayed under conditions of MW as it has been previously reported when target saliency was low (Töllner et al. 2011), during periods of attentional blink or shortly after task switches (Corriveau et al. 2012; Lagroix et al. 2015). Still, there was a substantial increase in response time (about 400 ms), when subjects reported to be “OFF” task which might have reflected lower sensory evidence, a delayed processing of the information provided by the N2pc, or could be caused by parallel interfering processes of MW. In fact, Lagroix et al. (2015) suspected that a response time increase caused by the attentional blink (about 300 ms) was not fully accounted for by N2pc latency differences (about 30 ms). However, later steps of information processing which might be impacted by MW—such as extraction of information or response planning—might play a role. Importantly, only when participants experienced MW (OFF task), the amplitude of the N2pc was positively correlated with performance. That is, a larger N2pc, typically associated with a stronger focusing onto the target and potentially reflecting better distractor suppression (Mazza et al. 2009; Donohue et al. 2016), might have partially compensated for the MW. Alternatively, it is reasonable to assume that the enhancement of the N2pc amplitude might not reflect a stronger selective distractor suppression but the participation of more neurons in the attention process. One might speculate that the attentional tuning is less strict and, hence, broader under MW with more (less selective) neurons responding to the attention focusing process, and in consequence leading to a larger N2pc. Since we have no baseline measure (MW without N2pc increase), it is difficult to determine how much the enhancement of the N2pc might have helped performance under MW. Still, at the between-subjects level, under MW, a higher N2pc amplitude was associated with a better performance speaking for a behavioral relevance of the N2pc increase.

When investigating MW, a major challenge is how to reliably capture phases of reduced focusing on the task. Frequently prompting thought probes during the course of the experiments will most likely discourage MW, hence, we chose to assess the participants mental state on only 20% of the trials. As a consequence, trial numbers are inherently limited for comparing neural responses between mental states. Furthermore, participants reported for the majority of trials (51%) to be “on task”, which might be caused by the perceptually rather demanding discrimination task, or also be influenced by participants trying to respond in a socially desirable way. Nevertheless, the markers of local sleep (SWA increase, HFA reduction) match participants self-reports with being “off the task” and might also provide future measures depending less on self-report.

Our critical conclusion is that MW is strongly linked to cortical dynamics associated with local sleep and that attentional resources needed for visual search are upregulated to circumvent restrictions caused by limited sensory evidence. Occipital HFA, which shows a strong stimulus response comparable to intracranial recordings, falls out when participants have the subjective impression of being off the task, commensurate with an increase in periods of SWA increase. Attentional decoupling as predicted for being off the task is expected to produce a decrease in the N2pc (Schad et al. 2012; Christoff et al. 2016). But reduced sensory evidence compels stronger attentional allocation to key features in the environment and hence a stronger target-distractor disambiguation during MW. Hence, these results indicate that MW does not lead to a global blackout of HFA but cortical regions generating the target-distractor disambiguation also flexibly react to internal distractions. These functional explanations indicate that expected input to visual stimulation is tracked and stronger reallocation of spatial attention is generated when sensory evidence is scarce, presumably by frontal cortical areas. In sum, we provide evidence that MW is strongly related to local sleep and establish a direct link between boosted attentional resources due to local sleep during waking.

## Notes

C.W., M.V.B., and S.D. conceived and designed the experiment. C.W. collected the MEG data. C.W., C.R., and S.D. analyzed the data, C.W., L.V., M.V.B., C.R., H.H., H.J.H, and S.D. interpreted the data. S.D., M.V.B., and C.W. wrote the manuscript. *Conflict of Interest*: None declared.

## Funding

This work was funded by Schwerpunkt Neuroforschung MW-21 LMSP 9-2012, funded by Land Sachsen-Anhalt. C.R. was funded by the Federal Ministry of Education and Research, Germany, grant number 13GW0095D.
